# Physicochemical Characteristics, Phenolic Profile, Antioxidant Potential, and Antimicrobial Activity of Bulgarian Summer Savory (*Satureja hortensis* L.)

**DOI:** 10.3390/cimb47121030

**Published:** 2025-12-10

**Authors:** Yulian Tumbarski, Magdalena Stoyanova, Petya Ivanova, Albena Parzhanova, Krastena Nikolova

**Affiliations:** 1 Department of Microbiology and Biotechnology, University of Food Technologies, 26 Maritsa Blvd., 4002 Plovdiv, Bulgaria; 2 Department of Analytical Chemistry and Physical Chemistry, University of Food Technologies, 26 Maritsa Blvd., 4002 Plovdiv, Bulgaria; magdalena.stoianova@abv.bg; 3 Department of Biochemistry and Nutrition, University of Food Technologies, 26 Maritsa Blvd., 4002 Plovdiv, Bulgaria; petia_ivanova_georgieva@abv.bg; 4 Department of Food Technologies, Institute of Food Preservation and Quality, Agricultural Academy, 154 Vasil Aprilov Blvd., 4002 Plovdiv, Bulgaria; albenadsp@abv.bg; 5 Department of Physics and Biophysics, Faculty of Pharmacy, Medical University—Varna, 9000 Varna, Bulgaria

**Keywords:** savory, summer savory, phenolic profile, antioxidant activity, antimicrobial activity

## Abstract

Summer savory (*Satureja hortensis* L.) is an annual herbaceous plant belonging to the *Lamiaceae* family and widely used as a culinary spice. The present research aimed to investigate the physicochemical characteristics, phenolic profile, antioxidant potential, and antimicrobial activity of ten summer savory samples (dried leaves and flowers) grown in different regions of Bulgaria. The physicochemical analyses of the dried plant material demonstrated that the ash content varied from 19.51 to 26.92%, proteins from 16.25 to 22.78%, and carbohydrates from 5.37 to 10.01%. The values of the total phenolic content (TPC) ranged from 1.10 to 4.83 mg GAE/g dw (aqueous savory extracts—ASE) and from 3.98 to 9.54 mg GAE/g dw (methanolic savory extracts—MSE). The values of the total flavonoid content (TFC) were from 0.08 to 0.29 mg QE/g dw (ASE) and from 0.73 to 1.23 mg QE/g dw (MSE). The investigated samples exhibited significant antioxidant activity, with values determined by the DPPH method varying between 35.01 and 59.93 mM TE/g dw (ASE) and between 51.75 and 91.85 mM TE/g dw (MSE). The values by the FRAP method ranged from 74.49 to 134.85 µmol Fe^2+^/g dw (ASE) and from 108.20 to 215.48 µmol Fe^2+^/g dw (MSE). The high-performance liquid chromatography (HPLC) analysis showed that rosmarinic acid was predominant in all tested samples (3.54–5.90 mg/g dw), whereas ferulic, caffeic and *p*-coumaric acids were detected in trace amounts. The HPLC analysis of organic acids revealed that ascorbic acid was present in higher concentration in all samples (0.35–0.98 mg/g dw) compared to malic acid, which was found in trace amounts. The antimicrobial activity test demonstrated that methanolic savory extracts showed moderate to high inhibitory activity against most of the microorganisms used (most pronounced against *Staphylococcus aureus* 6538P, with diameters of the inhibition zones from 20 to 30 mm), while aqueous savory extracts exhibited antifungal rather than antibacterial activity. Based on the results obtained, we can conclude that savory is a plant with potential for use in the pharmaceutical and agricultural sectors, in addition to its culinary applications.

## 1. Introduction

Summer savory (*Satureja hortensis* L.) is an annual herbaceous plant, belonging to the *Lamiaceae* family, and the most popular amongst the other varieties of savory, such as winter and autumn savory. The plant reaches a height of 30–60 cm and blooms between July and September with violet tubular flowers. Summer savory is native to the Mediterranean region and has been historically cultivated across adjacent warm areas of southeastern Europe and southwestern Asia. Over time, its cultivation spread widely through Europe, and today it is grown in many countries beyond its natural range. Summer savory is a plant widely used fresh or dried as a spice in the culinary world due to its strong aroma that imparts a pleasant flavor to various dishes. In the traditional Bulgarian cuisine, savory is one of the three ingredients in the spice mixture named “sharena sol” along with crimson chili and salt [1,2].

*S. hortensis* L. is an aromatic plant whose aerial parts are known for their rich phytochemical composition. It has been reported that they contain proteins, sugars, fats, fibers, minerals, vitamins (vitamin A, niacin), resins, tannins, triterpenic acids, essential oils, and others [2,3]. Summer savory is distinguished by its significant content of essential minerals for human health, such as iron (Fe): 242–726 mg/kg dw, potassium (K): 1.68–3.38 mg/kg dw, calcium (Ca): 1.08–2.84 mg/kg dw, phosphorus (P): 0.31–0.72 mg/kg dw, magnesium (Mg): 0.25–0.61 mg/kg dw, and sodium (Na): 0.007–0.013 mg/kg dw, which contribute to its high nutritional value and potential for application in food additives [4]. Summer savory essential oil comprises terpenes and terpenoids, such as monoterpenes (α-terpinene, γ-terpinene, *p*-cymene, α-phellandrene, α- and β-pinene, sabinene, α-thujene, limonene, terpineol, L-carvone), sesquiterpenes (β-bisabolene, caryophyllene oxide), and phenolic monoterpenoids (thymol, carvacrol). The phenolic compounds, which are detected in the summer savory extracts, include phenolic acids (rosmarinic, caffeic, isoferulic, chlorogenic), flavanones (naringenin), flavones (apigenin, apigenin-glucoside, luteolin, vitexin, apigetrin), flavonols (quercetin, isoquercitrin, quercitrin, kaempferol, astragalin), and coumarins (aesculin, aesculetin) [2]. It should be noted here that their amounts can vary widely depending on the plant cultivar (genotype), geographical region, environmental conditions, and agricultural measures applied during the cultivation [2] and harvest period [5], as well as post-harvest processing [6].

Some of the secondary metabolites of summer savory have potential as biologically active compounds that exhibit antimicrobial, antiseptic, antioxidant, anti-inflammatory, antiparasitic, hepatoprotective, anticancer, and other activities. For instance, carvacrol and thymol, contained in essential oil, demonstrate strong antibacterial, antifungal, anesthetic, anti-inflammatory, and antihelmintic effects [6]. Phenolic acids (particularly rosmarinic acid) and flavonoids are well known for their strong antioxidant activity and their contribution to the biological properties of *S. hortensis* L. [7]. Recent studies have revealed the potential of summer savory extracts, which are rich in rosmarinic acid, to prevent oxidative stress-related tissue damage, thereby exerting beneficial effects on the hepatic, renal, and testicular functions [8]. A study conducted by Muthukumar et al. [9] demonstrated that hydroalcoholic summer savory extract exhibited significant cardioprotective effects in isoproterenol-induced myocardial infarction in rats. Pre-treatment with the extract at a dose of 400 mg/kg significantly reduced cardiac damage by normalizing key biochemical markers, reducing oxidative stress, and preserving myocardial tissue structure. Folk medicine applications of summer savory have also been reported in the treatment of different cardiac, gastrointestinal (nausea, indigestion, diarrhea), muscular (cramps, muscle pain), and infectious disorders [10,11]. *S. hortensis* has been extensively studied for its pharmacological potential. Several studies have demonstrated its antinociceptive and anti-inflammatory effects [12], antigenotoxic activity against oxidative stress in rat lymphocytes [13], antioxidant, antidiabetic, and hypolipidemic properties [14,15], as well as cardiovascular protective effects by inhibition of blood platelet functions [16].

Beyond its nutritional value, recognized health-promoting properties, and potential pharmaceutical uses, savory has also been investigated for other practical applications such as food biopreservation. According to Nabigol [17], the application of water emulsions containing essential oils of three Iranian savory species on fresh strawberries inhibited the growth of plant pathogenic fungi *Rhizopus stolonifer*, *Penicillium digitatum*, *Aspergillus niger*, and *Botrytis cinerea*, and effectively prolonged the post-harvest fruit’s shelf life. Hosseini et al. [18] reported that the incorporation of summer savory essential oil in chitosan-based edible coatings prevented weight loss and maintained the vitamin C content and post-harvest quality of kumquat fruit (*Fortunella* sp.) during storage at 7 °C for 30 days.

Presently, the research on summer savory has primarily focused on the chemical composition, biological properties, and applications of its essential oil, whereas information on the plant and its various extracts remains limited, particularly with respect to Bulgarian-grown summer savory. In this context, the present study was undertaken to comprehensively investigate the physicochemical characteristics, phenolic profile, antioxidant potential, and antimicrobial activity of ten summer savory samples (dried leaves and flowers) cultivated in different regions of Bulgaria.

## 2. Materials and Methods

### 2.1. Materials

#### 2.1.1. Plant Material

**Figure 1:**
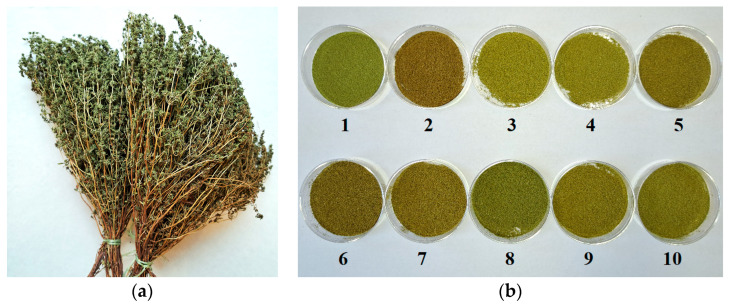
Dried aerial parts of *Satureja hortensis* L. in whole form ((**a**); sample 8) and ground form ((**b**); samples 1–10) were used for analyses.

Summer savory (*Satureja hortensis* L.) samples (dried leaves and flowers) from ten different locations in Bulgaria were purchased from private producers. The plant material was harvested during the flowering period (July–August 2025) and identified according to the Herbarium Academiae Scientiarum Bulgariae (Table 1). After harvesting, the aerial parts were shade-dried. Once dried, the leaves and flowers were manually stripped from the stems, which were discarded. The samples were put in brown paper bags, labeled, and stored at room temperature in darkness. The samples were finely ground by a blender and passed through a sieve (hole diameter = 0.5 mm) prior to analysis (Figure 1).

#### 2.1.2. Test Microorganisms

Five Gram-positive bacteria (*Bacillus subtilis* ATCC 6633, *Bacillus cereus* NCTC 11145, *Staphylococcus aureus* ATCC 6538P, *Listeria monocytogenes* NBIMCC 8632, *Enterococcus faecalis* RC-21), five Gram-negative bacteria (*Salmonella enteritidis* ATCC 13076, *Klebsiella pneumoniae* RC-20, *Escherichia coli* ATCC 25922, *Pseudomonas aeruginosa* ATCC 9027, *Proteus vulgaris* ATCC 6380), yeasts (*Candida albicans* NBIMCC 74), and four fungi (*Aspergillus niger* ATCC 1015, *Aspergillus flavus*, *Penicillium chrysogenum*, *Fusarium moniliforme* ATCC 38932) were selected for the antimicrobial activity test. The microbial strains used in this study were obtained from the collection of the Department of Microbiology and Biotechnology at the University of Food Technologies, Plovdiv, Bulgaria.

#### 2.1.3. Culture Media

Luria–Bertani agar medium with glucose (LBG agar)

LBG agar was used for the cultivation of test bacteria. A quantity of 50 g of LBG-solid substance mixture (containing 10 g tryptone, 5 g yeast extract, 10 g NaCl, 10 g glucose, and 15 g agar) was dissolved in 1 L of deionized water, pH 7.5 ± 0.2.

Malt extract agar (MEA)

MEA was used for the cultivation of test yeasts and fungi. A quantity of 50 g of the MEA-solid substance mixture (containing 30 g malt extract, 5 g mycological peptone, and 15 g agar) was dissolved in 1 L of deionized water, pH 5.4 ± 0.2.

The culture media were prepared according to the manufacturer’s instructions (Scharlab SL, Madrid, Spain) and autoclaved at 121 °C for 20 min (liquid phase) before use.

### 2.2. Methods

#### 2.2.1. Physicochemical Analyses

Moisture Content

Determination of moisture was performed using a KERN DAB 100-3 moisture balance analyzer (Kern&Sohn GmbH, Balingen, Germany) by heating a 1 g sample of ground plant material at 110 °C to a constant weight. The moisture content (%) was automatically recorded by the analyzer based on the difference between the initial weight and the weight after drying of the sample [19].

Protein Content

The protein content was determined by the nitrogen content of the samples according to the Kjeldahl nitrogen determination method using a BÜCHI K-365 Kjel Line automatic system (BÜCHI Labortechnik AG, Flawil, Switzerland) [19].

Ash Content and Carbohydrates

Ash content and carbohydrates were determined according to the Bulgarian State Standards [20,21].

#### 2.2.2. Preparation of Summer Savory Extracts

Two types of summer savory extracts (aqueous—ASE and methanolic—MSE) were prepared. For each extraction, 2 g of dried and ground savory material was macerated with 20 mL of deionized water or methanol in tightly sealed plastic tubes to minimize oxygen and light exposure. The mixtures were vortexed and then left to stand for 72 h at room temperature (22 ± 1 °C) in darkness, with occasional shaking to enhance solvent penetration. After extraction, the samples were filtered through Whatman No. 1 filter paper, and the final extract volume was recorded. Prior to analysis, the extracts were stored at 4 °C and, when necessary, further clarified by filtration through 0.45 μm PTFE membrane filters.

#### 2.2.3. Total Phenolic Content (TPC)

The total phenolic content of savory extracts was assessed according to the method of Ivanov et al. [22] using the Folin–Ciocalteu reagent (Sigma-Aldrich, St. Louis, MO, USA). The absorbance was measured at 765 nm, and the results were expressed as mg equivalents of gallic acid per gram of dry weight (mg GAE/g dw).

#### 2.2.4. Total Flavonoid Content (TFC)

The total flavonoid content of savory extracts was evaluated following the method described by Ivanov et al. [22]. The absorbance was measured at 415 nm, and the results were expressed as mg of quercetin equivalents per gram of dry weight (mg QE/g dw).

#### 2.2.5. Antioxidant Activity

DPPH Radical Scavenging Assay

The DPPH assay was performed by the method of Ivanov et al. [22] using DPPH (2,2-diphenyl-1-picrylhydrazyl) reagent (Sigma-Aldrich, St. Louis, MO, USA). The absorbance was measured at 517 nm, and the results were expressed as mM Trolox equivalents per gram of dry weight (mM TE/g dw).

Ferric-Reducing Antioxidant Power (FRAP) Assay

The FRAP assay was performed according to the method of Ivanov et al. [22] using 2,4,6-Tris(2-pyridyl)-s-triazine (TPTZ) (Sigma-Aldrich, St. Louis, MO, USA). The absorbance was measured at 593 nm, and the results were expressed as µmol Fe^2+^ per gram of dry weight (µmol Fe^2+^/g dw).

#### 2.2.6. High-Performance Liquid Chromatography (HPLC) Analysis of Phenolic Acids

For the quantification of phenolic acids, methanolic savory extracts were used. The phenolic acids were determined using an HPLC unit Elite LaChrome (VWR™ Hitachi, Tokyo, Japan) equipped with a diode array detector (DAD) as previously described [22]. Rosmarinic, ferulic, caffeic, and *p*-coumaric acids were detected at 320 nm. The results were expressed as mg/g dw.

#### 2.2.7. HPLC Analysis of Organic Acids

For the quantification of organic acids, aqueous savory extracts were used. The analysis was performed on the Elite LaChrome (Hitachi, Tokyo, Japan) HPLC system equipped with DAD [23]. The separation was conducted on a Discovery^®^ SH C18 column (25 cm  ×  4.6 mm, 5 μm) at 30 °C and isocratic elution with a mobile phase consisting of 25 mM KH_2_PO_4_ (pH = 2.4 with H_3_PO_4_). L-(+)-ascorbic acid was detected at 244 nm, while L-malic acid—at 210 nm. The results were expressed as mg/g dw.

Standards of phenolic and organic acids for HPLC analysis were purchased from Sigma-Aldrich (Saint Louis, MO, USA), all with a purity of ≥95.5%. The calibration and validation parameters for the standard compounds are presented in Table S1 (Supplementary Materials).

#### 2.2.8. Antimicrobial Activity Assay

The antimicrobial activity of ASE and MSE was assessed by the agar well diffusion method as previously described by Tumbarski et al. [24]. Antimicrobial activity was evaluated based on the diameter of inhibition zones (IZs) measured at 24 and 48 h post-incubation. Microorganisms were classified as sensitive (IZ ≥ 18 mm), moderately sensitive (IZ 12–18 mm), or resistant (IZ ≤ 12 mm or no visible zone). The antibiotics cefotaxime (against bacteria) and nystatin (against yeasts and fungi) at a concentration of 10 mg/mL were used as positive controls, while methanol and sterile distilled water served as negative controls.

#### 2.2.9. Statistical Analysis

The results from triplicate experiments were expressed as mean values ± standard deviation (±SD). One-way analysis of variance (ANOVA) was performed using the Statgraphics Centurion statistical program version XVI, 2009 (Stat Point Technologies, Inc., Warrenton, VA, USA). The mean differences were established by Fisher’s least significant difference test for paired comparison with a significance level of *p* ≤ 0.05. Pearson’s correlation coefficient (*r*^2^) was applied to assess the strength of the relationship between variables.

## 3. Results and Discussion

### 3.1. Physicochemical Characteristics

The results of the physicochemical analyses of the dried summer savory material are presented in Table 2. The data obtained demonstrated that the moisture content varied from 10.97% (sample 8) to 14.21% (sample 6), dry matter from 85.79% (sample 6) to 89.03% (sample 8), ash content from 19.51% (sample 8) to 26.92% (sample 9), proteins from 16.25 to 22.78%, and carbohydrates from 5.37% (sample 1) to 10.01% (sample 8).

Despite its widespread use as a culinary and medical plant, *S. hortensis* has not been extensively investigated in terms of its physicochemical characteristics. A study conducted by Alexa et al. [25] on the proximate composition of the dry plant material of *S. hortensis* from the western part of Romania reported a moisture content of 7% and protein content of 11.83%, both of which were lower than the values found in our study. According to other scientific data, the ash content of summer savory was 7.5%, while the protein content was 15% [3].

### 3.2. Total Phenolic Content, Total Flavonoid Content, and Antioxidant Activity of Summer Savory Extracts

The results for the TPC and the TFC of the summer savory extracts are presented in Table 3. As seen from the results, the TPC values varied from 1.10 mg GAE/g dw (sample 7) to 4.83 mg GAE/g dw (sample 1) for the aqueous extracts (ASE), and from 3.98 mg GAE/g dw (sample 6) to 9.54 mg GAE/g dw (sample 9) for the methanolic extracts (MSE). The TFC values ranged from 0.08 mg QE/g dw (sample 8) to 0.29 mg QE/g dw (sample 2) for ASE, and from 0.73 mg QE/g dw (sample 2) to 1.23 mg QE/g dw (sample 1) for MSE. Overall, the methanolic extracts exhibited higher values of both TPC and TFC compared to the aqueous extracts, indicating that the type of solvent used for extraction significantly influenced the yield of biologically active compounds. Despite its lower polarity compared to water, methanol often provides higher extraction efficiency for phenolic compounds due to its optimal solvent characteristics and better ability to penetrate plant tissues [26].

The results for the antioxidant activity of the summer savory samples are presented in Table 4. As shown by the data, the values determined by the DPPH assay varied from 35.01 mM TE/g dw (sample 9) to 59.93 mM TE/g dw (sample 6) for ASE, and from 51.75 mM TE/g dw (sample 2) to 91.85 mM TE/g dw (sample 1) for MSE. The values determined by the FRAP assay ranged from 74.49 µmol Fe^2+^/g dw (sample 9) to 134.85 µmol Fe^2+^/g dw (sample 6) for ASE, and from 108.20 µmol Fe^2+^/g dw (sample 2) to 215.48 µmol Fe^2+^/g dw (sample 9) for MSE. Similarly to the results for TPC and TFC discussed above, the methanolic extracts also exhibited higher antioxidant potential, as assessed by the DPPH and FRAP methods, compared to the aqueous extracts, which can be explained by the higher efficiency of methanol as an extraction solvent. These findings also support the positive relationship between polyphenolic content and antioxidant capacity.

It has been found that the antioxidant activity correlates strongly with the levels of phenolic compounds, supporting the general understanding that they are the major contributors to antioxidant potential [27]. The relationships between total phenolic content (TPC), total flavonoid content (TFC), and antioxidant activity (determined by DPPH and FRAP assays) for summer savory extracts are presented in Table 5 and Figures S1–S10 (Supplementary Materials). The results demonstrated a positive linear correlation (*r*^2^) among the tested parameters. The highest correlation coefficient was observed between the antioxidant activity values measured by the DPPH and FRAP assays, particularly for the methanolic extracts (MSE, *r*^2^ = 0.8624) and aqueous extracts (ASE, *r*^2^ = 0.9686), followed by the correlations between TFC and DPPH (*r*^2^ = 0.6989) and TFC and FRAP (*r*^2^ = 0.6576) for MSE.

The polyphenolic and flavonoid contents, along with the antioxidant activity of *S. hortensis*, have been widely studied due to their significant biological properties. Ergün [28] reported that the methanolic extract of *S. hortensis* from Erzurum province, Turkey, exhibited TPC and TFC values of 40.85 mg GAE/g and 26.52 mg QE/g, respectively, which were higher than those found in our research, while the antioxidant activity, as determined by the DPPH method, was 45.24 μg TE/mL. Predescu et al. [29] found that the ethanolic extract of Romanian summer savory exhibited values for TPC and TFC of 12.14 mg GAE/g dw and 7.49 mg catechin equivalents (CE)/g dw, respectively, whereas the antioxidant activity showed values of IC_50_ = 17.94 μg/mL (DPPH assay) and 39.55 μmol Fe^2+^/g dw (FRAP assay). Yesiloğlu et al. [30] investigated the TPC, TFC, and antioxidant capacity of acetone, ethanolic, and water extracts obtained from leaves and flowers of *S. hortensis* collected in Turkey. In contrast to our results, the authors reported that the water extract contained higher levels of phenolics and flavonoids compared to the ethanolic and acetone extracts, which correlated with enhanced antioxidant activity. Mohamed et al. [31] examined the biological activity of *S. hortensis* from Egypt and stated that the 80% ethanolic extract demonstrated TPC and TFC values of 26.04 mg GAE/g dw and 4.297 mg QE/g, respectively, which were higher than those obtained in our study for the aqueous and methanolic extracts. The DPPH radical scavenging activity was reported as 98.01%. The TPC and TFC values of *S. hortensis* ethanolic extract, as reported by Bahramikia et al. [32], were 128 mg GAE/g and 88.5 mg CE/g of dried extract, respectively. The antioxidant activity, assessed using the FRAP assay, was 645.3 μM Fe^2+^/mg of dried extract. A strong correlation between the DPPH radical scavenging activity and the TPC (*r*^2^ = 0.798) and between DPPH radical scavenging activity and TFC (*r*^2^ = 0.894) of *S. hortensis* samples was reported by Bimbiraitė-Survilienė et al. [33].

The TPC and TFC of wild mountain savory (*Satureja montana* L.) from Bulgaria, examined by Vrancheva [34], were highest in the 40% water-ethanolic extract, with values of 29.58 mg GAE/g dw and 8.45 mg QE/g dw, respectively, compared to the 70% water-ethanolic and aqueous extracts. This extract also exhibited the highest antioxidant activity—233.86, 478.54, 310.64, and 674.18 mM TE g/dw—evaluated by four spectrophotometric methods: DPPH, 2,2′-azino-bis(3-ethylbenzothiazoline-6-sulfonic acid) (ABTS), FRAP, and cupric reducing antioxidant capacity (CUPRAC), respectively. Another study performed by Vrancheva et al. [35] on a wild-growing population of *S. montana* L. from Bulgaria reported similar values of TPC and TFC—36.54 mg GAE/g dw and 11.11 mg QE/g dw, respectively—as well as comparable antioxidant capacity, with values determined by the DPPH, ABTS, FRAP, and CUPRAC assays of 308.42, 542.09, 381.22, and 723.53 mM TE/g dw, respectively.

### 3.3. High-Performance Liquid Chromatography (HPLC) Analysis of Phenolic Acids and Organic Acids of Summer Savory Extracts

In the present study, HPLC/DAD was used for the identification and quantification of phenolic and organic acids. Four phenolic acids (rosmarinic, ferulic, caffeic, and *p*-coumaric) and two organic acids (ascorbic and malic) were identified, and their contents (expressed as mg/g dw) are presented in Table 6. The peaks corresponding to the identified phenolic and organic acids are shown in the HPLC chromatograms presented in Figures S11 and S12 (Supplementary Materials).

Rosmarinic acid was the predominant phenolic compound identified in all *S. hortensis* samples, with concentrations ranging from 3.54 mg/g dw (sample 3) to 5.90 mg/g dw (sample 4). Conversely, ferulic, caffeic, and *p*-coumaric acids were detected in trace amounts (<0.1 mg/g dw). These results were consistent with those obtained in some previous studies. Using HPLC analysis, Chkhikvishvili et al. [36] identified rosmarinic and ferulic acids as the major phenolic compounds in the ethanolic extract of *S. hortensis* from Georgia. Vrancheva [34] reported values of rosmarinic acid of 3.11, 3.76, and 1.35 mg/g dw in 70% water–ethanolic, 40% water–ethanolic, and water extracts, respectively, of wild-growing *S. montana* populations from Bulgaria. Similarly, Shekarchi et al. [37] observed rosmarinic acid levels between 1.2 and 16.0 mg/g dw across various *Satureja* species. Boroja et al. [8] reported a concentration of rosmarinic acid of 24.9 mg/g of dry extract, while caffeic, isoferulic, *p*-coumaric, sinapic, and chlorogenic acids were found at levels at least 20 times lower than that of rosmarinic acid. It is important to note that the rosmarinic acid is a key antioxidant compound in summer savory, and its content can vary significantly due to both intrinsic and extrinsic factors, including soil and climatic conditions, developmental stage, harvest time, and storage conditions [37]. It is well established that rosmarinic acid, a caffeic acid ester, is a common secondary metabolite found in *Lamiaceae* species, exhibiting a wide range of biological activities [38]. As a natural phenolic compound, rosmarinic acid has been reported to possess anti-carcinogenic [39], anti-angiogenic [40], antidepressant [41], anti-inflammatory [42], anti-allergic [43], antimicrobial [44], neuroprotective [45], and HIV-1-inhibiting properties [46].

In our study, the ascorbic acid (vitamin C) content determined by the HPLC assay ranged from 0.35 mg/g dw (sample 3) to 0.98 mg/g dw (sample 2), while malic acid was detected in trace amounts (<0.1 mg/g dw). The ascorbic acid levels were considerably higher than those previously reported for summer savory (23.37 mg/100 g dw). When compared to other *Lamiaceae* spices such as rosemary (16.94 mg/100 g dw) and thyme (15.7 mg/100 g dw), the studied summer savory samples exhibited a relatively higher concentration of ascorbic acid [47]. The ascorbic acid content in summer savory can vary significantly depending on environmental and agronomic factors. In this regard, Dzida et al. [48] reported that ascorbic acid levels were higher when sowing was performed in April rather than in May, and during the first harvest (July) compared to the second one (August).

### 3.4. Antimicrobial Activity of Summer Savory Extracts

The results of the antimicrobial activity test of summer savory extracts against various microorganisms, along with the positive controls, are presented in Table 7 and Table 8. As shown, both the aqueous (ASE) and methanolic (MSE) extracts exhibited low to high inhibitory activity, with inhibition zone diameters (IZs) ranging from 8 to 28 mm. Regarding antibacterial effects, the ASE demonstrated weak inhibitory activity only against *P. vulgaris* ATCC 6380 (IZs = 8–10 mm). In contrast, samples 3, 5, 6, 7, 8, 9, and 10 exhibited the strongest inhibition (IZs ≥ 18 mm) against *A. niger* ATCC 1015 (IZs = 18–28 mm). Samples 1 and 7 showed the highest activity against *A. flavus* (IZs = 20 mm), while samples 2 and 6 were most effective against *P. chrysogenum* (IZs = 19–22 mm).

The methanolic savory extracts (MSE) exhibited both antibacterial and antifungal activity (Table 8). The highest antimicrobial activity was observed in all samples against *S. aureus* ATCC 6538P (IZs = 20–28 mm), while against the remaining Gram-positive, as well as Gram-negative bacteria, the MSE displayed low to moderate inhibitory activity. Regarding antifungal activity, all samples exhibited low to moderate inhibitory effect on the yeasts *C. abicans* NBIMCC 74 and the fungal strains *A. niger* ATCC 1015, *A. flavus*, *P. chrysogenum*, and *F. moniliforme* ATCC 38932. Methanol and sterile distilled water, which were used as negative controls, did not inhibit the growth of the test microorganisms (data not presented).

Based on the results obtained, it can be concluded that the aqueous savory extracts demonstrated stronger antifungal than antibacterial effect, whereas the methanolic savory extracts exhibited higher antibacterial but limited antifungal effectiveness.

To date, a limited number of studies have been conducted on the antimicrobial properties of various summer savory extracts. Predescu et al. [29] determined that the ethanolic extract of Romanian summer savory exhibited significant antimicrobial activity against *S. aureus* ATCC 9144 (IZ = 28.1 mm), which was consistent with our results. The authors stated that the extract was also effective against *Staphylococcus epidermidis* ATCC 12228 (IZ = 23.3 mm), *E. coli* ATCC 25922 (IZ = 25.6 mm), *S. enteritidis* ATCC 13076 (IZ = 16.4 mm), and *Salmonella typhimurium* ATCC 14028 (IZ = 11 mm). Popovici et al. [49] observed lower antibacterial and no antifungal activity of the hydro-alcoholic extract of *S. hortensis* using the disk diffusion assay. The extract, tested at a concentration of 250 μg/disk, showed weak antibacterial activity only against *Shigella flexneri* ATCC 12022, *S. typhimurium* ATCC 14028, and *Streptococcus pyogenes* ATCC 19615 (IZs = 7–8 mm). No antifungal activity was recorded against *C. albicans* ATCC 10231 and *Candida parapsilosis* ATCC 22019. Özcan and Al-Juhaimi [50] investigated summer savory extract (obtained using a solvent mixture of 90% methanol, 9% water, and 1% acetic acid) and found that at concentrations of 0.1%, 0.3%, and 0.5%, the extract exhibited significant inhibitory activity against the fungal strains *A. niger*, *Alternaria alternata*, and *Aspergillus parasiticus* NRRL 2999, as the antifungal effect increased with the extract concentration and persisted throughout the incubation period. The antifungal activity of *S. hortensis* was confirmed in a study by Dikbas et al. [51], who tested its methanolic extract against *Aspergillus flavus*. Using the disk-diffusion assay, Adiguzel et al. [52] demonstrated that the methanolic extract of *S. hortensis* exhibited inhibitory activity against several bacterial strains, including *Acinetobacter baumannii* A8, *Acinetobacter lwoffii* F1, *Bacillus macerans* A199, *B. subtilis* ATCC 6633, *B. subtilis* A57, *K. pneumoniae* F3, *P. vulgaris* A161, and *Staphylococcus epidermidis* A233. Ethanolic, methanolic, and dichloromethane extracts of *S. hortensis* from Erzincan province (Turkey) exhibited antimicrobial activity against nine bacterial and fungal strains, with the strongest effect observed for all extracts against *A. baumannii* at concentrations ranging from 25 to 800 µg/mL [53]. Hexane, methanol, and their mixture extracts of *S. hortensis* demonstrated potential as seed disinfectants against plant pathogenic bacteria, effectively reducing disease severity and enhancing germination and seedling growth in infected lettuce and tomato seeds. The hexane–methanol extract mixture at a concentration of 2.5 mg/mL was effective against *Xanthomonas axanopodis* pv. *vitians*, without exhibiting phytotoxic effects. These findings suggest that *S. hortensis* can be used as a seed disinfectant and as a natural pesticide for the management of plant bacterial diseases [54].

The antimicrobial effects of *S. hortensis* extracts may be attributed to the activity of their polyphenolic constituents. According to Daglia [55], polyphenols such as phenolic acids exert inhibitory activity mainly by disrupting the cytoplasmic membrane, increasing its permeability, and causing leakage of intracellular contents. Additional mechanisms include inhibition of key bacterial enzymes, induction of oxidative stress, and metal-ion chelation, all of which impair microbial metabolism. Therefore, it can be suggested that the antimicrobial activity of summer savory extracts is related to the mechanisms previously described for polyphenol-rich plant extracts.

## 4. Conclusions

The results of this study demonstrated that summer savory (*Satureja hortensis* L.) grown in different regions of Bulgaria possessed valuable physicochemical properties, a rich phenolic profile, and significant antioxidant potential. Methanolic extracts contained higher concentrations of bioactive compounds compared to aqueous extracts, which correlated with stronger antimicrobial activity, particularly against certain bacterial strains. HPLC analysis confirmed the presence of key phenolic and organic acids, with rosmarinic and ascorbic acids being the most abundant in all samples. These findings are consistent with, and in some cases exceed, previously reported values, highlighting the regional variability of bioactive compound content. Based on these characteristics, summer savory can be considered a valuable plant not only for culinary purposes but also for pharmaceutical, nutraceutical, and agricultural applications. Future studies should explore the influence of cultivation conditions, harvest time, and extraction methods on the bioactive profile to further optimize its potential uses.

## Figures and Tables

**Table 1:** Origin of the summer savory (*Satureja hortensis* L.) samples.

Savory Sample No.	Village/Town	District/Region	GPS Coordinates	Altitude, m	Voucher No.
1	Kyustendil	Kyustendil	42°28′ N 22°69′ E	525	SOM 179935
2	Mramor	Sofia	42°79′ N 23°27′ E	526	SOM 179936
3	Yundola	Pazardzhik	42°03′ N 23°52′ E	1400	SOM 179937
4	Stara Zagora	Stara Zagora	42°43′ N 25°63′ E	214	SOM 179938
5	Pazardzhik	Pazardzhik	42°19′ N 24°33′ E	211	SOM 179939
6	Avramovo	Blagoevgrad	42°03′ N 23°81′ E	1295	SOM 179940
7	Radomir	Pernik	42°55′ N 22°95′ E	764	SOM 179941
8	Krichim	Plovdiv	42°04′ N 24°46′ E	253	SOM 179942
9	Pleven	Pleven	43°42′ N 24°61′ E	116	SOM 179943
10	Belovitsa	Plovdiv	42°25′ N 24°31′ E	293	SOM 179944

**Table 2:** Physicochemical characteristics of summer savory samples.

Savory Sample No.	Moisture, %	Dry matter, %	Ash, %	Proteins, %	Carbohydrates, %
1	12.18 ± 1.28 ^bcd^	87.82 ± 1.28 ^bcd^	23.15 ± 0.09 ^g^	22.78 ± 0.41 ^a^	5.37 ± 0.00 ^h^
2	11.12 ± 0.48 ^d^	88.88 ± 0.48 ^d^	23.38 ± 0.09 ^f^	16.25 ± 0.33 ^e^	6.96 ± 0.01 ^f^
3	11.88 ± 0.21 ^bcd^	88.12 ± 0.21 ^bcd^	21.40 ± 0.09 ^h^	18.00 ± 0.40 ^bc^	6.27 ± 0.00 ^g^
4	11.53 ± 0.30 ^cd^	88.47 ± 0.30 ^cd^	25.43 ± 0.09 ^c^	16.37 ± 0.48 ^e^	8.20 ± 0.01 ^e^
5	11.67 ± 0.46 ^cd^	88.33 ± 0.46 ^cd^	25.72 ± 0.09 ^b^	17.24 ± 0.29 ^cd^	8.17 ± 0.01 ^e^
6	14.21 ± 0.40 ^a^	85.79 ± 0.40 ^a^	24.49 ± 0.09 ^e^	17.04 ± 0.28 ^de^	8.44 ± 0.01 ^d^
7	12.81 ± 0.35 ^abc^	87.19 ± 0.35 ^abc^	25.80 ± 0.09 ^b^	16.92 ± 0.42 ^de^	9.67 ± 0.01 ^b^
8	10.97 ± 0.01 ^d^	89.03 ± 0.01 ^d^	19.51 ± 0.09 ^i^	22.43 ± 0.70 ^a^	10.01 ± 0.01 ^a^
9	13.29 ± 1.51 ^ab^	86.71 ± 1.51 ^ab^	26.92 ± 0.09 ^a^	17.77 ± 0.01 ^bcd^	8.29 ± 0.01 ^de^
10	12.10 ± 0.46 ^bcd^	87.90 ± 0.46 ^bcd^	24.79 ± 0.09 ^d^	18.42 ± 0.03 ^b^	8.77 ± 0.01 ^c^

^a–i^: Means in a column without a common letter differ significantly (*p* < 0.05).

**Table 3:** Total phenolic content (TPC) and total flavonoid content (TFC) of summer savory extracts.

Savory Sample No.	TPC (mg GAE/g dw)		TFC (mg QE/g dw)	
	ASE	MSE	ASE	MSE
1	4.83 ± 0.08 ^b,A^	4.96 ± 0.35 ^e,A^	0.17 ± 0.00 ^e,B^	1.23 ± 0.11 ^a,A^
2	2.83 ± 0.33 ^e,B^	4.45 ± 0.10 ^e,A^	0.29 ± 0.01 ^a,B^	0.73 ± 0.01 ^b,A^
3	2.57 ± 0.28 ^ef,B^	7.50 ± 0.19 ^cd,A^	0.16 ± 0.00 ^f,B^	0.99 ± 0.16 ^abc,A^
4	4.11 ± 0.12 ^c,B^	6.84 ± 0.30 ^d,A^	0.27 ± 0.00 ^b,B^	1.08 ± 0.09 ^ab,A^
5	2.29 ± 0.42 ^fg,B^	8.82 ± 0.46 ^b,A^	0.14 ± 0.00 ^h,B^	0.89 ± 0.13 ^bc,A^
6	2.99 ± 0.00 ^c,A^	3.98 ± 0.20 ^f,B^	0.25 ± 0.00 ^c,B^	0.83 ± 0.18 ^bc,A^
7	1.10 ± 0.17 ^a,A^	8.60 ± 0.48 ^b,B^	0.14 ± 0.00 ^h,B^	0.81 ± 0.12 ^bc,A^
8	2.78 ± 0.12 ^e,B^	8.88 ± 0.27 ^ab,A^	0.08 ± 0.00 ^i,B^	1.11 ± 0.12 ^ab,A^
9	1.83 ± 0.05 ^g,B^	9.54 ± 0.39 ^a,A^	0.23 ± 0.00 ^d,B^	1.01 ± 0.12 ^abc,A^
10	3.37 ± 0.12 ^d,B^	7.72 ± 0.10 ^c,A^	0.15 ± 0.00 ^g,B^	0.96 ± 0.25 ^abc,A^

^a–i^: Means in a column without a common letter differ significantly (*p* < 0.05). ^A,B^: Means in a row (for a particular method) without a common letter differ significantly (*p* ≤ 0.05). ASE—aqueous savory extracts; MSE—methanolic savory extracts.

**Table 4:** Antioxidant activity of summer savory extracts.

Savory Sample No.	DPPH (mM TE/g dw)		FRAP (µmol Fe^2+^/g dw)	
	ASE	MSE	ASE	MSE
1	35.63 ± 0.25 ^e,B^	91.85 ± 0.57 ^a,A^	86.64 ± 0.36 ^f,B^	196.52 ± 0.27 ^b,A^
2	43.09 ± 0.67 ^d,B^	51.75 ± 0.63 ^h,A^	89.15 ± 0.62 ^e,B^	108.20 ± 0.45 ^i,A^
3	35.85 ± 0.21 ^e,B^	83.50 ± 0.62 ^c,A^	83.85 ± 1.22 ^g,B^	154.59 ± 0.88 ^g,A^
4	42.94 ± 0.68 ^d,B^	81.20 ± 0.64 ^d,A^	93.04 ± 0.37 ^d,B^	179.96 ± 1.37 ^d,A^
5	35.69 ± 0.33 ^e,B^	87.17 ± 0.95 ^b,A^	81.53 ± 0.33 ^h,B^	185.13 ± 2.68 ^c,A^
6	59.93 ± 1.09 ^a,A^	62.55 ± 0.76 ^g,A^	134.85 ± 0.48 ^a,B^	136.48 ± 0.15 ^h,A^
7	49.86 ± 0.36 ^b,B^	78.48 ± 1.25 ^e,A^	107.89 ± 0.53 ^b,B^	164.42 ± 1.19 ^f,A^
8	35.07 ± 0.73 ^e,B^	76.42 ± 0.35 ^f,A^	81.44 ± 0.36 ^h,B^	162.09 ± 0.42 ^f,A^
9	35.01 ± 0.61 ^e,B^	83.35 ± 0.75 ^c,A^	74.49 ± 0.33 ^i,B^	215.48 ± 0.75 ^a,A^
10	45.47 ± 0.68 ^c,B^	84.76 ± 0.37 ^c,A^	95.94 ± 0.46 ^c,B^	171.22 ± 2.36 ^e,A^

^a–i^: Means in a column without a common letter differ significantly (*p* < 0.05). ^A,B^: Means in a row (for a particular method) without a common letter differ significantly (*p* ≤ 0.05). ASE—aqueous savory extracts; MSE—methanolic savory extracts.

**Table 5:** Correlation between the total phenolic content (TPC), the total flavonoid content (TFC) and the antioxidant activity of summer savory extracts.

Methods	Correlation, r2	
	MSE	ASE
TPC/DPPH	0.5672	0.4649
TPC/FRAP	0.5822	0.4681
TFC/DPPH	0.6989	0.3591
TFC/FRAP	0.6576	0.2488
DPPH/FRAP	0.8624	0.9686

ASE—aqueous savory extracts; MSE—methanolic savory extracts.

**Table 6:** Phenolic acid and organic acid contents of summer savory samples were determined by HPLC analysis.

Savory Sample No.	Phenolic Acids, mg/g dw				Organic Acids, mg/g dw	
	Rosmarinic	Ferulic	Caffeic	*p*-Coumaric	Ascorbic	Malic
1	5.07 ± 0.10 ^cd,A^	0.04 ± 0.00 ^d,C^	0.07 ± 0.00 ^a,B^	0.005 ± 0.00 ^c,D^	0.51 ± 0.02 ^c,A^	0.004 ± 0.00 ^c,B^
2	3.98 ± 0.10 ^f,A^	0.04 ± 0.00 ^d,C^	0.07 ± 0.01 ^a,B^	0.006 ± 0.00 ^b,D^	0.98 ± 0.06 ^a,A^	0.005 ± 0.00 ^b,B^
3	3.54 ± 0.10 ^g,A^	0.03 ± 0.00 ^e,C^	0.04 ± 0.00 ^d,B^	0.005 ± 0.00 ^c,D^	0.35 ± 0.05 ^e,A^	0.003 ± 0.00 ^d,B^
4	5.90 ± 0.20 ^a,A^	0.07 ± 0.01 ^a,B^	0.07 ± 0.01 ^a,B^	0.005 ± 0.00 ^c,C^	0.49 ± 0.01 ^c,A^	0.004 ± 0.00 ^c,B^
5	5.28 ± 0.20 ^bc,A^	0.05 ± 0.00 ^c,B^	0.05 ± 0.00 ^c,B^	0.004 ± 0.00 ^d,C^	0.52 ± 0.07 ^c,A^	0.004 ± 0.00 ^c,B^
6	4.43 ± 0.10 ^e,A^	0.06 ± 0.01 ^b,B^	0.04 ± 0.00 ^d,C^	0.005 ± 0.00 ^c,D^	0.42 ± 0.02 ^d,A^	0.006 ± 0.00 ^a,B^
7	5.14 ± 0.10 ^bc,A^	0.06 ± 0.01 ^b,B^	0.06 ± 0.01 ^b,B^	0.006 ± 0.00 ^b,C^	0.51 ± 0.02 ^c,A^	0.006 ± 0.00 ^a,B^
8	4.81 ± 0.10 ^d,A^	0.04 ± 0.00 ^d,C^	0.07 ± 0.00 ^a,B^	0.008 ± 0.00 ^a,D^	0.41 ± 0.01 ^de,A^	0.004 ± 0.00 ^c,B^
9	5.79 ± 0.20 ^a,A^	0.05 ±.0.00 ^c,C^	0.06 ± 0.01 ^b,B^	0.006 ± 0.00 ^b,D^	0.78 ± 0.01 ^b,A^	0.005 ± 0.00 ^b,B^
10	5.42 ± 0.20 ^b,A^	0.06 ± 0.01 ^b,B^	0.06 ± 0.00 ^b,B^	0.006 ± 0.00 ^b,C^	0.41 ± 0.02 ^de,A^	0.006 ± 0.00 ^a,B^

^a–g^: Means in a column without a common letter differ significantly (*p* < 0.05). ^A–D^: Means in a row (for phenolic/organic acids) without a common letter differ significantly (*p* ≤ 0.05).

**Table 7:** Antimicrobial activity of the aqueous summer savory extracts (ASE).

Test MO *	Diameter of the Inhibition Zones, mm											
	Aqueous Summer Savory Extracts (100 mg/mL)										Controls	
	1	2	3	4	5	6	7	8	9	10	C **	N ***
*B. s.*	-	-	-	-	-	-	-	-	-	-	27 ± 0.00	n.a.
*B. c.*	-	-	-	-	-	-	-	-	-	-	38 ± 0.00	n.a.
*S. a.*	-	-	-	-	-	-	-	-	-	-	45 ± 0.00	n.a.
*L. m.*	-	-	-	-	-	-	-	-	-	-	37 ± 0.00	n.a.
*E. f.*	-	-	-	-	-	-	-	-	-	-	-	n.a.
*S. e.*	-	-	-	-	-	-	-	-	-	-	40 ± 0.00	n.a.
*K. p.*	-	-	-	-	-	-	-	-	-	-	40 ± 0.00	n.a.
*E. c.*	-	-	-	-	-	-	-	-	-	-	30 ± 0.00	n.a.
*P. a.*	-	-	-	-	-	-	-	-	-	-	20 ± 0.00	n.a.
*P. v.*	10 ± 0.00	8 ± 0.00	8 ± 0.00	8 ± 0.00	10 ± 0.00	8 ± 0.00	8 ± 0.00	8 ± 0.00	10 ± 0.00	8 ± 0.00	45 ± 0.00	n.a.
*C. a.*	-	15 ± 0.00	13 ± 0.00	15 ± 0.00	-	-	-	13 ± 0.00	10 ± 0.00	10 ± 0.00	n.a.	12 ± 0.00
*A. n.*	-	-	20 ± 0.00	15 ± 0.00	21 ± 0.00	18 ± 0.00	28 ± 0.00	20 ± 0.00	21 ± 0.00	22 ± 0.00	n.a.	-
*A. f.*	20 ± 0.00	-	-	14 ± 0.00	15 ± 0.00	-	20 ± 0.00	10 ± 0.00	10 ± 0.00	-	n.a.	-
*P. c.*	-	22 ± 0.00	15 ± 0.00	-	-	19 ± 0.00	16 ± 0.00	14 ± 0.00	12 ± 0.00	10 ± 0.00	n.a.	-
*F. m.*	10 ± 0.00	15 ± 0.00	13 ± 0.00	-	-	15 ± 0.00	14 ± 0.00	10 ± 0.00	-	12 ± 0.00	n.a.	12 ± 0.00

*B. s.*—*B. subtilis* ATCC 6633; *B. c.*—*B. cereus* NCTC 11145; *S. a.*—*S. aureus* ATCC 6538P; *L. m.*—*L. monocytogenes* NBIMCC 8632; *E. f.*—*E. faecalis* RC-21; *S. e.*—*S. enteritidis* ATCC 13076; *K. p.*—*K. pneumoniae* RC-20; *E. c.*—*E. coli* ATCC 25922; *P. a.*—*P. aeruginosa* ATCC 9027; *P. v.*—*P. vulgaris* ATCC 6380; *C. a.*—*C. albicans* NBIMCC 74; *A. n.*—*A. niger* ATCC 1015; *A. f.*—*A. flavus*, *P. c.*—*P. chrysogenum*, *F. m.*—*F. moniliforme* ATCC 38932. * MO—microorganisms; ** Cefotaxime (10 mg/mL); *** Nystatin (10 mg/mL); n.a.—not applied.

**Table 8:** Antimicrobial activity of the methanolic summer savory extracts (MSE).

Test MO *	Diameter of the Inhibition Zones, mm											
	Methanolic Summer Savory Extracts (100 mg/mL)										Controls	
	1	2	3	4	5	6	7	8	9	10	C **	N ***
*B. s.*	11 ± 0.00	11 ± 0.00	11 ± 0.00	11 ± 0.00	11 ± 0.00	11 ± 0.00	11 ± 0.00	11 ± 0.00	12 ± 0.00	10 ± 0.00	27 ± 0.00	n.a.
*B. c.*	11 ± 0.00	12 ± 0.00	12 ± 0.00	12 ± 0.00	12 ± 0.00	12 ± 0.00	11 ± 0.00	11 ± 0.00	12 ± 0.00	11 ± 0.00	38 ± 0.00	n.a.
*S. a.*	20 ± 0.00	20 ± 0.00	20 ± 0.00	20 ± 0.00	20 ± 0.00	20 ± 0.00	20 ± 0.00	28 ± 0.00	28 ± 0.00	25 ± 0.00	45 ± 0.00	n.a.
*L. m.*	12 ± 0.00	15 ± 0.00	12 ± 0.00	12 ± 0.00	12 ± 0.00	11 ± 0.00	11 ± 0.00	12 ± 0.00	11 ± 0.00	12 ± 0.00	37 ± 0.00	n.a.
*E. f.*	10 ± 0.00	12 ± 0.00	10 ± 0.00	10 ± 0.00	10 ± 0.00	10 ± 0.00	11 ± 0.00	10 ± 0.00	11 ± 0.00	10 ± 0.00	-	n.a.
*S. e.*	10 ± 0.00	12 ± 0.00	10 ± 0.00	10 ± 0.00	11 ± 0.00	11 ± 0.00	10 ± 0.00	11 ± 0.00	11 ± 0.00	10 ± 0.00	40 ± 0.00	n.a.
*K. p.*	10 ± 0.00	10 ± 0.00	10 ± 0.00	10 ± 0.00	10 ± 0.00	10 ± 0.00	9 ± 0.00	9 ± 0.00	10 ± 0.00	10 ± 0.00	40 ± 0.00	n.a.
*E. c.*	12 ± 0.00	12 ± 0.00	12 ± 0.00	12 ± 0.00	12 ± 0.00	12 ± 0.00	11 ± 0.00	13 ± 0.00	12 ± 0.00	12 ± 0.00	30 ± 0.00	n.a.
*P. a.*	12 ± 0.00	13 ± 0.00	13 ± 0.00	12 ± 0.00	12 ± 0.00	12 ± 0.00	13 ± 0.00	13 ± 0.00	12 ± 0.00	12 ± 0.00	20 ± 0.00	n.a.
*P. v.*	8 ± 0.00	8 ± 0.00	9 ± 0.00	9 ± 0.00	10 ± 0.00	10 ± 0.00	10 ± 0.00	10 ± 0.00	10 ± 0.00	10 ± 0.00	45 ± 0.00	n.a.
*C. a.*	15 ± 0.00	15 ± 0.00	13 ± 0.00	13 ± 0.00	14 ± 0.00	15 ± 0.00	12 ± 0.00	10 ± 0.00	10 ± 0.00	10 ± 0.00	n.a.	12 ± 0.00
*A. n.*	15 ± 0.00	15 ± 0.00	13 ± 0.00	12 ± 0.00	15 ± 0.00	13 ± 0.00	15 ± 0.00	15 ± 0.00	12 ± 0.00	12 ± 0.00	n.a.	-
*A. f.*	15 ± 0.00	15 ± 0.00	15 ± 0.00	12 ± 0.00	13 ± 0.00	16 ± 0.00	15 ± 0.00	12 ± 0.00	12 ± 0.00	12 ± 0.00	n.a.	-
*P. c.*	10 ± 0.00	10 ± 0.00	10 ± 0.00	10 ± 0.00	10 ± 0.00	10 ± 0.00	10 ± 0.00	12 ± 0.00	12 ± 0.00	10 ± 0.00	n.a.	-
*F. m.*	12 ± 0.00	13 ± 0.00	13 ± 0.00	12 ± 0.00	13 ± 0.00	11 ± 0.00	11 ± 0.00	13 ± 0.00	12 ± 0.00	11 ± 0.00	n.a.	12 ± 0.00

*B. s.*—*B. subtilis* ATCC 6633; *B. c.*—*B. cereus* NCTC 11145; *S. a.*—*S. aureus* ATCC 6538P; *L. m.*—*L. monocytogenes* NBIMCC 8632; *E. f.*—*E. faecalis* RC-21; *S. e.*—*S. enteritidis* ATCC 13076; *K. p.*—*K. pneumoniae* RC-20; *E. c.*—*E. coli* ATCC 25922; *P. a.*—*P. aeruginosa* ATCC 9027; *P. v.*—*P. vulgaris* ATCC 6380; *C. a.*—*C. albicans* NBIMCC 74; *A. n.*—*A. niger* ATCC 1015; *A. f.*—*A. flavus*, *P. c.*—*P. chrysogenum*, *F. m.*—*F. moniliforme* ATCC 38932. * MO—microorganisms; ** Cefotaxime (10 mg/mL); *** Nystatin (10 mg/mL); n.a.—not applied.

## Data Availability

The original contributions presented in this study are included in the article/Supplementary Materials. Further inquiries can be directed to the corresponding authors.

## Supplementary Materials

The following supporting information can be downloaded at https://www.mdpi.com/article/10.3390/cimb47121030/s1.
