# Supplementary material for: Physicochemical Characteristics, Phenolic Profile, Antioxidant Potential, and Antimicrobial Activity of Bulgarian Summer Savory (Satureja hortensis L.)
Source: Curr Issues Mol Biol. 2025 Dec 10;47(12):1030. doi: 10.3390/cimb47121030 (PMC12731340; doi:10.3390/cimb47121030)
Supplement: Supplementary file 1 [file cimb-47-01030-s001.zip › cimb-4016673-supplementary.pdf]

Article

Physicochemical characteristics, phenolic profile, antioxidant potential, and antimicrobial activity of Bulgarian summer savory (*Satureja hortensis* L.)

Yulian Tumbarski [1,*], Magdalena Stoyanova [2], Petya Ivanova [3], Albena Parzhanova [4] and Krastena Nikolova [5,*]

[1] Department of Microbiology and Biotechnology, University of Food Technologies, 26 Maritsa Blvd., 4002 Plovdiv, Bulgaria; tumbarski@abv.bg (Y.T.)
[2] Department of Analytical Chemistry and Physical Chemistry, University of Food Technologies, 26 Maritsa Blvd., 4002 Plovdiv, Bulgaria; magdalena.stoianova@abv.bg (M.S.)
[3] Department of Biochemistry and Nutrition, University of Food Technologies, 26 Maritsa Blvd., 4002 Plovdiv, Bulgaria; petia_ivanova_georgieva@abv.bg (P.I.)
[4] Department of Food Technologies, Institute of Food Preservation and Quality, Agricultural Academy, 154 Vasil Aprilov Blvd., 4002 Plovdiv, Bulgaria; albenadsp@abv.bg (A.P.)
[5] Department of Physics and Biophysics, Faculty of Pharmacy, Medical University - Varna, 9000 Varna, Bulgaria; Krastena.Nikolova@mu-varna.bg (K.N.)
* Correspondence: tumbarski@abv.bg; Krastena.Nikolova@mu-varna.bg

Abstract

Summer savory (*Satureja hortensis* L.) is an annual herbaceous plant, belonging to the *Lamiaceae* family and widely used as a culinary spice. The present research aimed to investigate the physicochemical characteristics, phenolic profile, antioxidant potential, and antimicrobial activity of ten summer savory samples (dried leaves and flowers) grown in different regions of Bulgaria. The physicochemical analyses of the dried plant material demonstrated that the ash content varied from 19.51 to 26.92 %, proteins – from 16.25 to 22.78 %, and carbohydrates – from 5.37 to 10.01 %. The values of the total phenolic content (TPC) ranged from 1.10 to 4.83 mg GAE/g dw (aqueous savory extracts - ASE) and from 3.98 to 9.54 mg GAE/g dw (methanolic savory extracts - MSE). The values of the total flavonoid content (TFC) were from 0.08 to 0.29 mg QE/g dw (ASE) and from 0.73 to 1.23 mg QE/g dw (MSE). The investigated samples exhibited significant antioxidant activity, with values determined by the DPPH method varied between 35.01 and 59.93 mM TE/g dw (ASE) and between 51.75 and 91.85 mM TE/g dw (MSE). The values by the FRAP method ranged from 74.49 to 134.85 μmol Fe^{2+}/g dw (ASE) and from 108.20 to 215.48 μmol Fe^{2+}/g dw (MSE). The high-performance liquid chromatography (HPLC) analysis showed that rosmarinic acid was predominant in all tested samples (3.54–5.90 mg/g dw), whereas ferulic, caffeic and *p*-coumaric acids were detected in trace amounts. The HPLC analysis of organic acids revealed that ascorbic acid was presented in higher concentration in all samples (0.35–0.98 mg/g dw) compared to malic acid, which was found in trace amounts. The antimicrobial activity test demonstrated that methanolic savory extracts showed moderate to high inhibitory activity against most of the microorganisms used (most pronounced against *Staphylococcus aureus* 6538P with diameter of the inhibition zones from 20 to 30 mm), while aqueous savory extracts exhibited antifungal rather than antibacterial activity. Based on the results obtained, we can conclude that savory is a plant with potential for use in the pharmaceutical and agricultural sectors, in addition to its culinary applications.

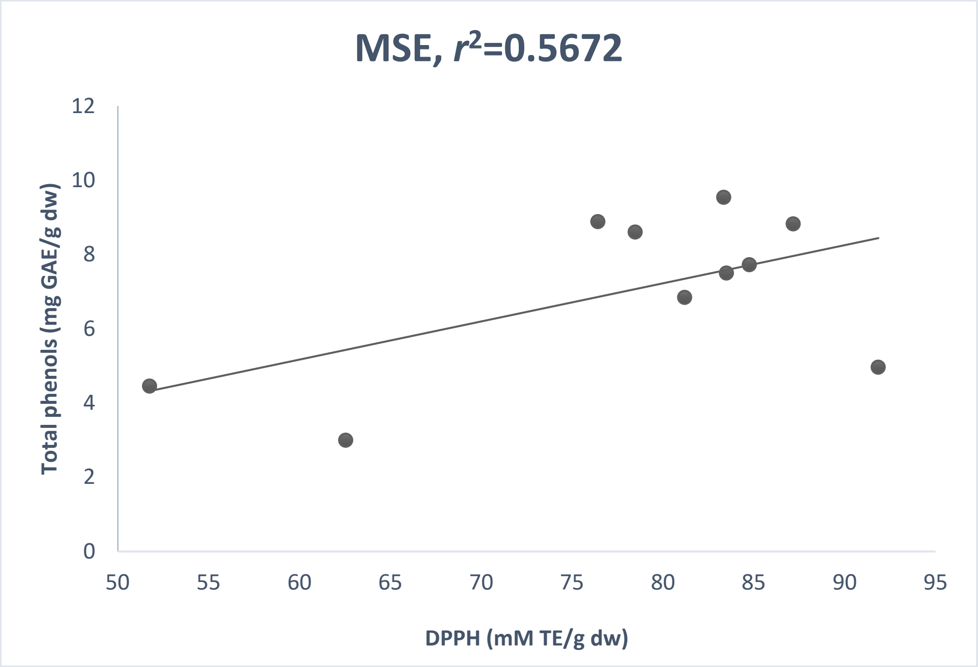

Fig. S1. Linear correlation between the total phenolic content (TPC) and antioxidant activity (DPPH assay) values of methanolic summer savory extracts (MSE).

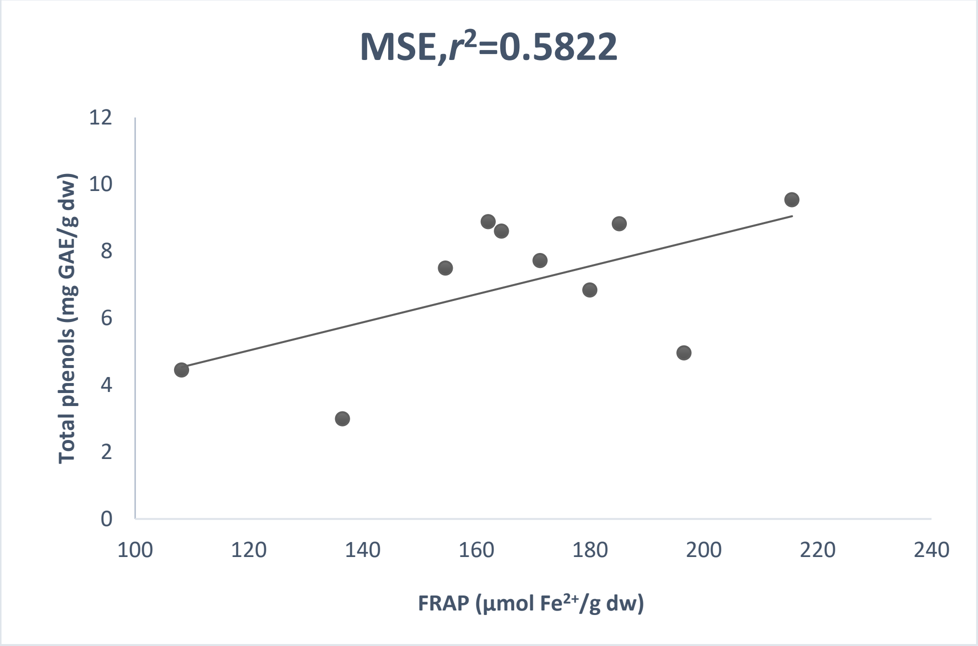

Fig. S2. Linear correlation between the total phenolic content (TPC) and antioxidant activity (FRAP assay) values of methanolic summer savory extracts (MSE).

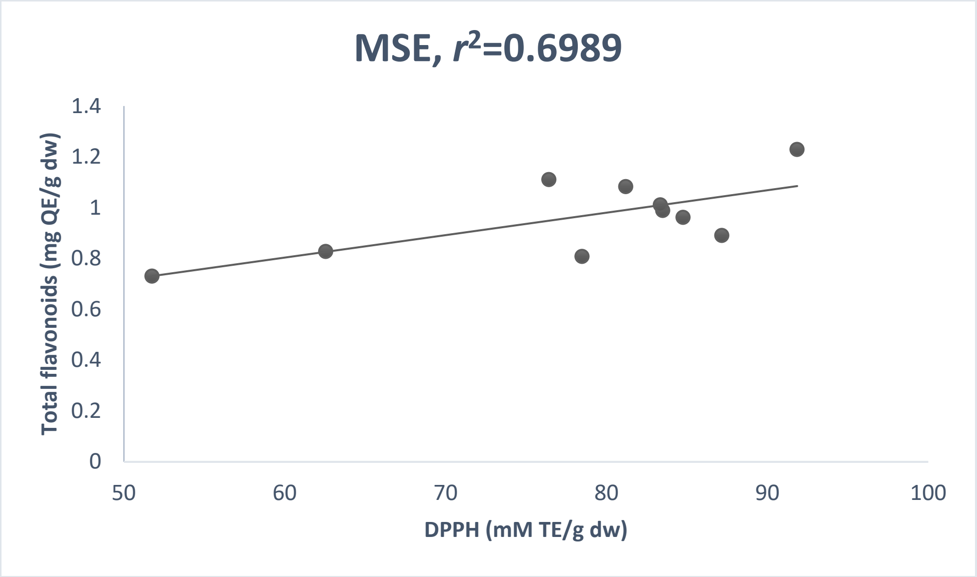

Fig. S3. Linear correlation between the total flavonoid content (TFC) and antioxidant activity (DPPH assay) values of methanolic summer savory extracts (MSE).

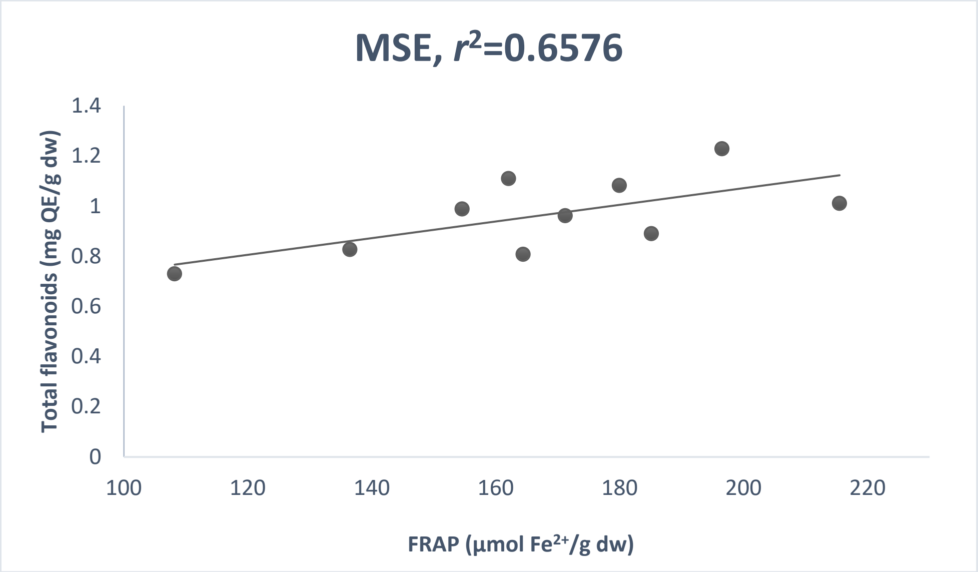

Fig. S4. Linear correlation between the total flavonoid content (TFC) and antioxidant activity (FRAP assay) values of methanolic summer savory extracts (MSE).

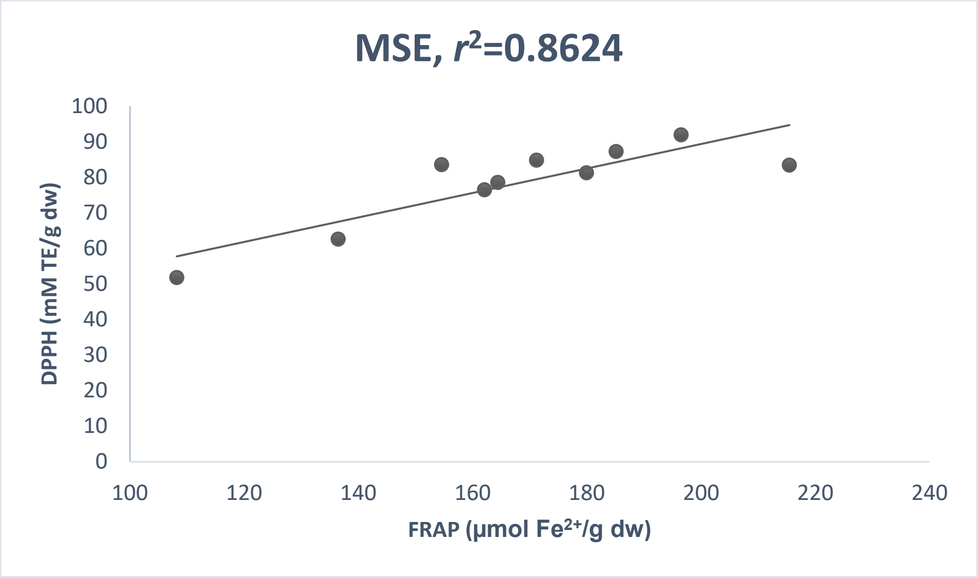

Fig. S5. Linear correlation between antioxidant activity values determined by DPPH and FRAP assays of methanolic summer savory extracts (MSE).

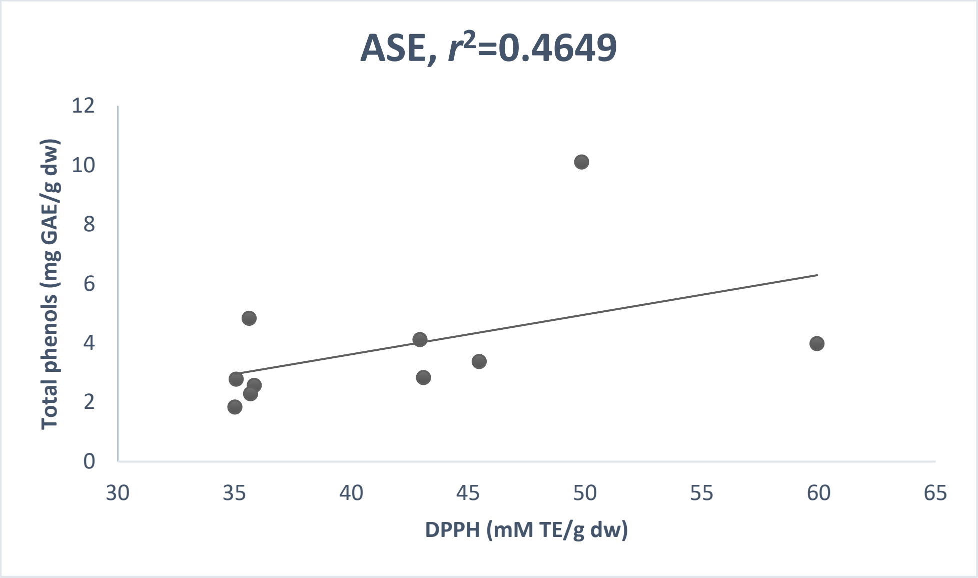

Fig. S6. Linear correlation between the total phenolic content (TPC) and antioxidant activity (DPPH assay) values of aqueous summer savory extracts (ASE).

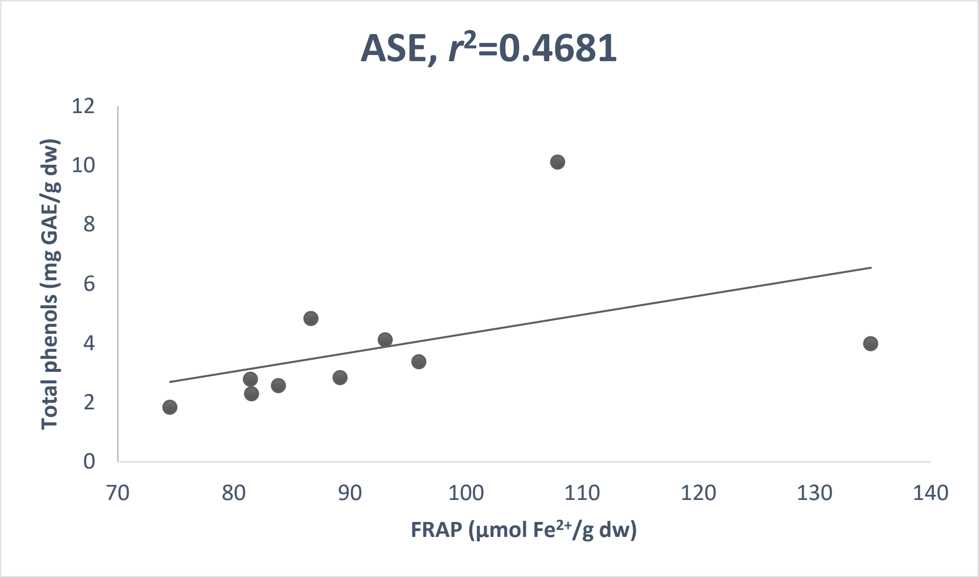

Fig. S7. Linear correlation between the total phenolic content (TPC) and antioxidant activity (FRAP assay) values of aqueous summer savory extracts (ASE).

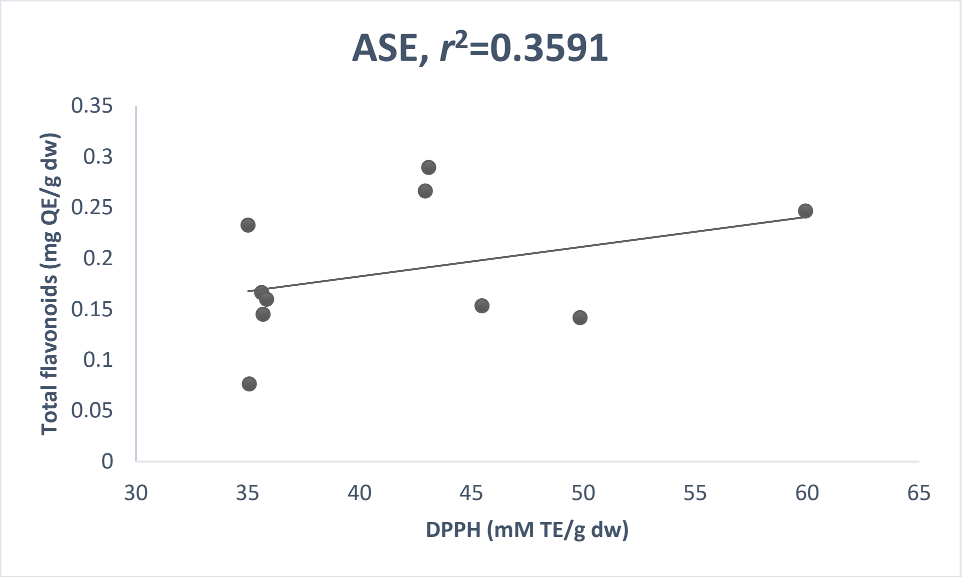

Fig. S8. Linear correlation between the total flavonoid content (TFC) and antioxidant activity (DPPH assay) values of aqueous summer savory extracts (ASE).

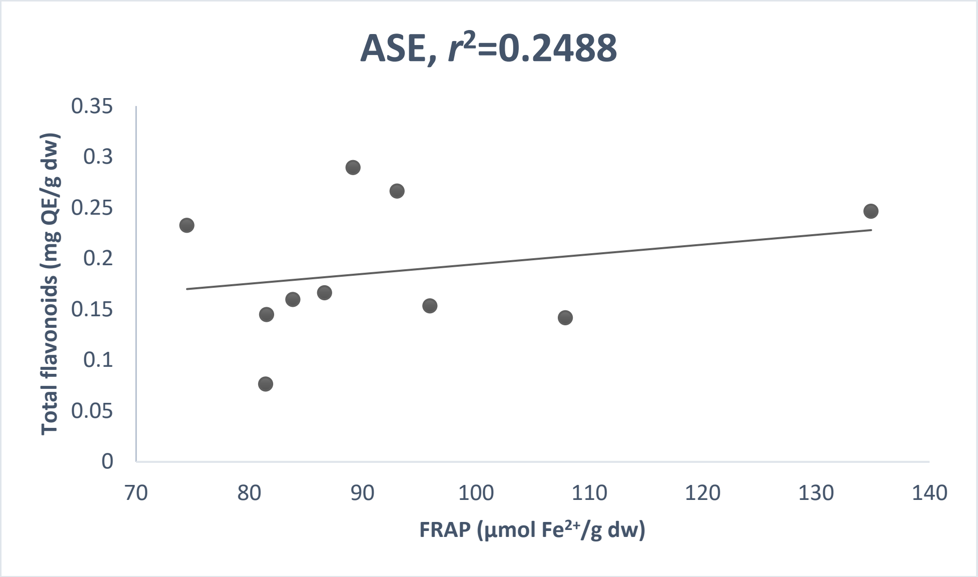

Fig. S9. Linear correlation between the total flavonoid content (TFC) and antioxidant activity (FRAP assay) values of aqueous summer savory extracts (ASE).

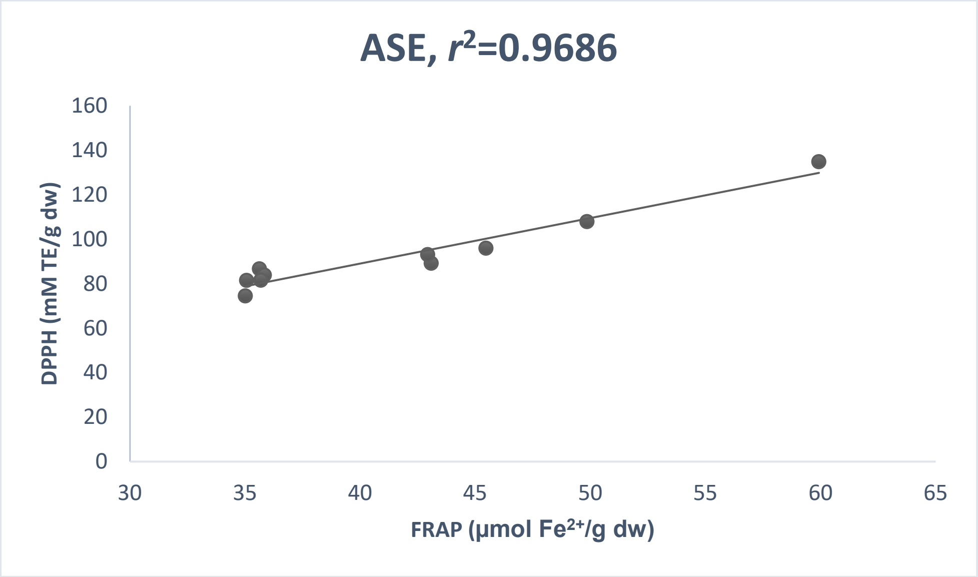

Fig. S10. Linear correlation between antioxidant activity values determined by DPPH and FRAP assays of aqueous summer savory extracts (ASE).

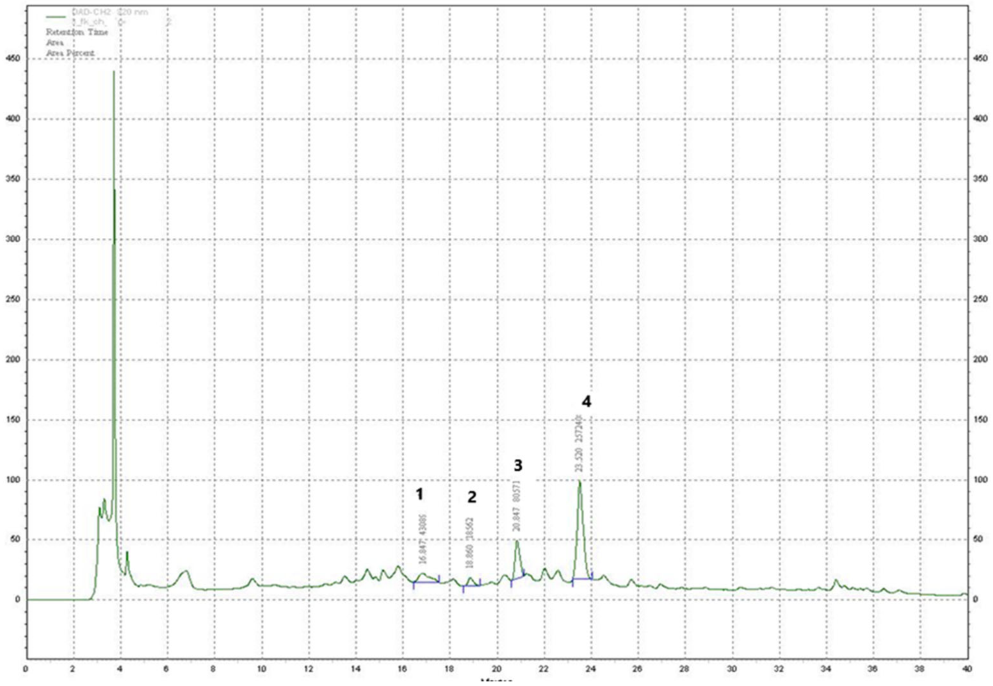

Fig. S11. Representative HPLC chromatogram of methanolic summer savory extract (MSE) monitored at 320 nm, showing the identified phenolic acids: (1) caffeic acid, (2) ferulic acid, (3) *p*-coumaric acid, (4) rosmarinic acid.

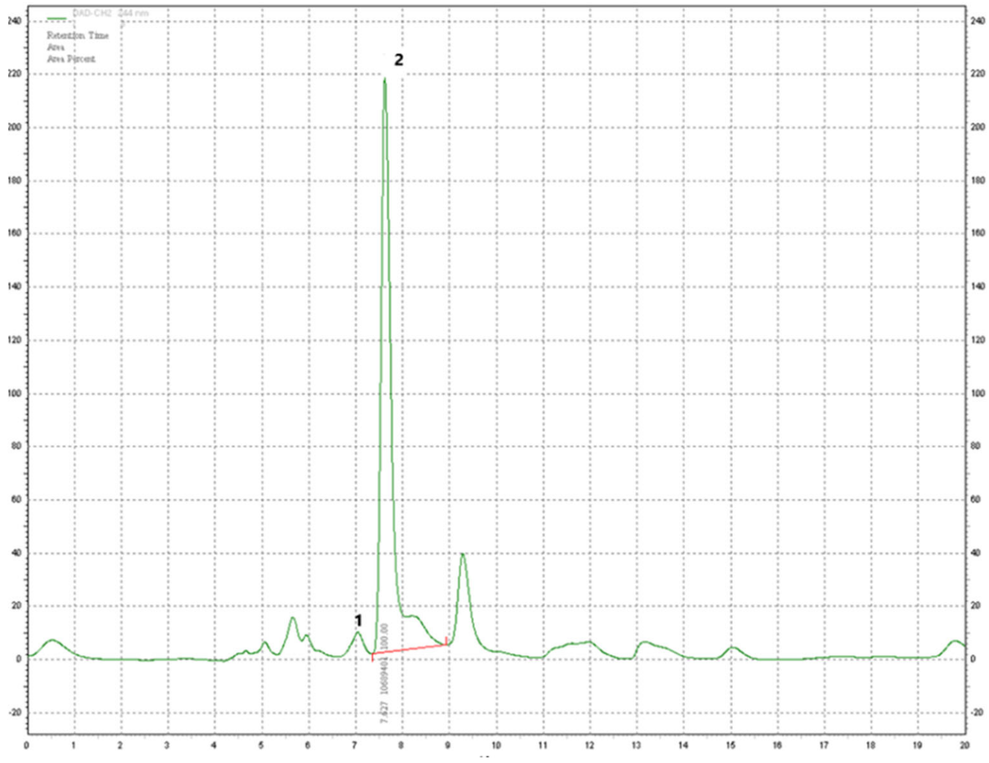

Fig. S12. Representative HPLC chromatogram of aqueous summer savory extract (ASE) monitored at 210 and 244nm, showing the identified organic acids: (1) L-malic acid, (2) L-(+)-ascorbic acid.

Table S1. Calibration and validation parameters for standard compounds.

Compound	Calibration range, μg/mL	Regression equation (y = ax + b)	R^2	LOD	LOQ	Intra-day precision (%RSD, n)	Inter-day precision (%RSD, n)	Recovery (%) (n, 3 levels)
Rosmarinic acid	10-100	Y =339408.X+523342	0.9997	1.80	5.94	2.2 n=6	3.2 n=6	98.9 n=6
Ferulic acid	10-100	Y=48798.X+914983	0.9874	1.60	5.28	1.8 n=6	2.0 n=6	99.2 n=6
Caffeic acid	10-100	Y=628288.X-427587	0.9900	1.90	6.27	2.2 n=6	3.1 n=6	98.8 n=6
p-coumaric acid	10-100	Y=665271.X-1775277	0.9980	1.20	3.96	2.0 n=6	2.9 n=6	99.3 n=6
Ascorbic acid	50-500	Y=462004162.X+5312351	0.9898	5.20	17.16	2.4 n=6	3.3 n=6	98.9 n=6
Malic acid	100-500	Y=71289289.X+2068826	0.9994	20.00	66.00	1.2 n=6	2.1 n=6	98.6 n=6
